# Temporal dynamics of trematode intermediate snail host environmental DNA in small water body habitats

**DOI:** 10.1017/S0031182021001104

**Published:** 2021-10

**Authors:** Rhys Aled Jones, Chelsea N. Davis, Dewi Llyr Jones, Fiona Tyson, Emma Davies, David Cutress, Peter M. Brophy, Michael T. Rose, Manod Williams, Hefin Wyn Williams

**Affiliations:** 1 Institute of Biological, Environmental and Rural Sciences, Aberystwyth University, Aberystwyth, UK; 2 Coleg Cambria, Llysfasi, Ruthin Road, Ruthin, Denbighshire, UK; 3 Tasmanian Institute of Agriculture, University of Tasmania, Sandy Bay, TAS, Australia

**Keywords:** *Calicophoron daubneyi*, environmental DNA, eDNA, *Fasciola hepatica*, fluke, *Galba truncatula*, generalized estimation equation, intermediate snail host, small water bodies, trematode

## Abstract

Environmental DNA (eDNA) surveying has potential to become a powerful tool for sustainable parasite control. As trematode parasites require an intermediate snail host that is often aquatic or amphibious to fulfil their lifecycle, water-based eDNA analyses can be used to screen habitats for the presence of snail hosts and identify trematode infection risk areas. The aim of this study was to identify climatic and environmental factors associated with the detection of *Galba truncatula* eDNA. Fourteen potential *G. truncatula* habitats on two farms were surveyed over a 9-month period, with eDNA detected using a filter capture, extraction and PCR protocol with data analysed using a generalized estimation equation. The probability of detecting *G. truncatula* eDNA increased in habitats where snails were visually detected, as temperature increased, and as water pH decreased (*P* < 0.05). Rainfall was positively associated with eDNA detection in watercourse habitats on farm A, but negatively associated with eDNA detection in watercourse habitats on farm B (*P* < 0.001), which may be explained by differences in watercourse gradient. This study is the first to identify factors associated with trematode intermediate snail host eDNA detection. These factors should be considered in standardized protocols to evaluate the results of future eDNA surveys.

## Graphical abstract

**Figure:**
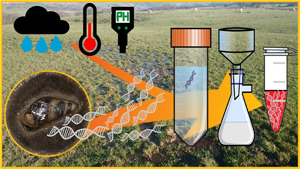

## Introduction

Environmental DNA (eDNA) surveys have become powerful tools to identify and monitor the presence of species in aquatic environments (Thomsen and Willerslev, 2015). Water-based eDNA analysis techniques have been developed and applied to detect rare and invasive species and infectious pathogens in aquatic environments, which have led to changes in policy and management (Bass *et al*., 2015; Thomsen and Willerslev, 2015). The application of eDNA analysis has the potential to become an effective tool in sustainable parasite control (Bass *et al*., 2015). Recent research has identified spatial patterns in trematode parasite eDNA with the aim of informing strategies to reduce transmission (Hashizume *et al*., 2017; Sengupta *et al*., 2019) and to evaluate parasite infection intensity in host populations (Huver *et al*., 2015). However, due to the complex nature of trematode lifecycles where multiple stages including eggs and miracidia which cannot infect the final host are found in the environment and are sources of eDNA, detection of trematode eDNA may not be an accurate measure of parasite prevalence in final host populations or transmission risk in the area (Jones *et al*., 2018). For trematodes to infect their final hosts, an intermediate snail host must first be infected, where the trematode will multiply asexually before developing into an infective stage. Therefore, identifying areas where these intermediate snail host species are present is essential to evaluate trematode epidemiology and inform control strategies.

In recent years, eDNA capture and detection protocols have been developed to detect intermediate snail host eDNA, including that of *Galba truncatula* (Jones *et al*., 2018; Davis *et al*., 2020), *Austropeplea tomentosa* (Rathinasamy *et al*., 2018; Rathinasamy *et al*., 2021), *Oncomelania hupensis* (Fornillos *et al*., 2019) and *Bulinus truncatus* (Mulero *et al*., 2020). As these snails are often present at low densities and are difficult to find and differentiate from non-intermediate host snails, eDNA surveys can outperform traditional physical detection methods (Jones *et al*., 2018) as has been shown for other organisms (Wilcox *et al*., 2016). However, eDNA surveys can be imperfect, and multiple factors may contribute to false-negative or even false-positive results, including climatic and environmental factors (Harrison *et al*., 2019), as well as limitations in sampling and analysis protocols (Goldberg *et al*., 2016). It is vital to mitigate against these issues, and site occupancy models can be built to rationally evaluate the probability of detecting target species' eDNA in a test sample based on multiple characteristics of the habitat sampled. These site characteristics can include area, water pH and temperature, recent climatic conditions, the number of replicates and volume of water sampled amongst others (Schmidt *et al*., 2013; Smith and Goldberg, 2020). However, at present there is limited data evaluating the temporal dynamics of intermediate snail host eDNA in relation to environmental and climatic factors, especially in small water body habitats.

In this study, a long-term survey of *G. truncatula* eDNA was performed across 14 small water habitats over a 9-month period. *Galba truncatula* is the main intermediate host snail of *C. daubneyi* and *F. hepatica* (Jones *et al*., 2015; Jones *et al*., 2017), a parasite that is estimated to cost the global livestock production and food industries €2.5 billion annually, and infects up to 17 million humans each year (European-Commisson, 2012). Due to growing concerns over anthelmintic resistance and residues (Kelley *et al*., 2016) and the impact of climate change in extending *F. hepatica*'s geographical and seasonal ranges (Fox *et al*., 2011), alternative control strategies are of increasing interest to livestock producers worldwide. The leading alternative strategies aim to limit contact between livestock and intermediate snail host in order to negate lifecycle success (Beesley *et al*., 2018). Developing accurate eDNA surveying tools and protocols will identify *G. truncatula* habitats on farmland and inform the successful application of alternative control strategies. Therefore, the objective of this study was to identify environmental and climatic factors associated with the detection of *G. truncatula* eDNA in small water body habitats.

## Methods

### Study design and eDNA analysis

**Fig. 1.**
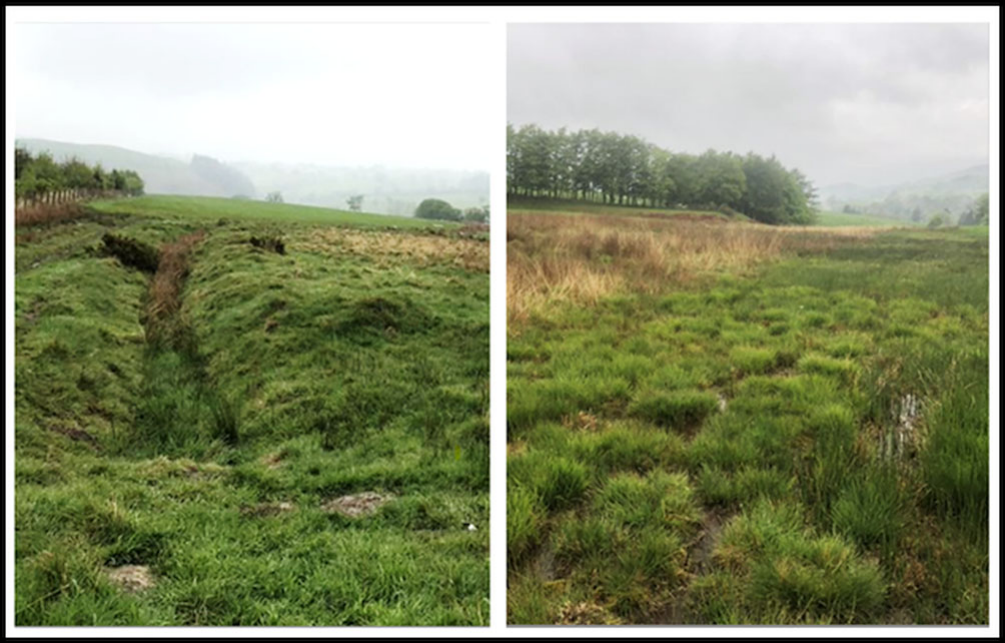
Example of a watercourse habitat (left) and pasture habitat (right), both from Farm B in this study.

Water samples were collected and analysed from 14 potential *G. truncatula* habitats on two farms. Eight habitats were located on Farm A (Aberystwyth University, Ceredigion, Wales, UK; Latitude 52°25′55.60″N, Longitude 4°1′8.85″W) and six habitats were located on Farm B (Llysfasi College, Ruthin, Wales, UK; Latitude 53°3′44.95″N, Longitude 3°16′25.98″W). Both farms had a history of *F. hepatica* and/or *C. daubneyi* infections in livestock. Habitats on both farms were selected for the study based on their physical suitability to harbour *G. truncatula* snails, with all habitats containing standing or flowing water for periods during the summer months, bare mud surfaces and *Juncaceae* spp. which are indicator plant species for *G. truncatula* habitats (Dreyfuss *et al*., 2015). These habitats were categorized as either being watercourses, defined as a channel of moving water <1.5 m in width, or pasture habitats, defined as an area of stagnant water pooled on pasture (Fig. 1). Habitat elevation ranged from 25 to 150 m on Farm A and 260 to 340 m on Farm B. There is no known hydrological connectivity between the habitats on both farms and the individual habitats are considered independent sampling sites. Further information regarding each study habitat, including a breakdown of habitat type, can be seen in Table 1. The study aimed to collect water samples from each habitat at weekly intervals between 21 March 2019 and 13 December 2019 on Farm A, and between 10 June 2019 and the 26 November 2019 on Farm B. However, samples were only collected if sufficient water was present to satisfy the sampling protocol. Four individuals collected water samples, all of which were trained and routinely supervised by one individual to ensure sample collection consistency.

**Table 1.** The environmental features of 14 habitats and the number of samples collected in each during the study

Farm	Habitat ID	Latitude	Longitude	Habitat type	Gradient (%)	Confirmed *G. truncatula* presence	*N* samples collected
A	1	53° 2′45.99″N	3°15′47.23″W	Watercourse	1.9	Yes	33
	2	53° 2′53.73″N	3°15′41.59″W	Pasture	–	Yes	2
	3	53° 2′41.46″N	3°15′21.85″W	Pasture	–	Yes	33
	4	53° 2′40.95″N	3°15′16.02″W	Pasture	–	No	6
	5	53° 2′37.24″N	3°15′15.60″W	Pasture	––	No	3
	6	53° 2′32.30″N	3°15′28.60″W	Pasture	–	No	7
	7	52°25′29.23″N	4°3′6.77″W	Watercourse	1.8	Yes	28
	8	52°25′27.37″N	4°3′8.42″W	Watercourse	1.6	No	33
B	9	52°25′16.94″N	4°3′3.20″W	Pasture	–	Yes	12
	10	52°25′29.63″N	4°2′54.36″W	Pasture	–	No	12
	11	52°25′31.00″N	4°1′53.19″W	Watercourse	9.8	Yes	13
	12	52°25′35.83″N	4°1′33.64″W	Watercourse	11.7	Yes	13
	13	52°25′38.37″N	4°1′31.85″W	Watercourse	10.4	Yes	13
	14	52°25′20.24″N	4°1′28.04″W	Watercourse	6.3	Yes	13

The number of *G. truncatula* snails present in each habitat was recorded during each sampling visit following a 10 min search of each habitat. Climate data for Farm A were obtained from the Plas Gogerddan weather station, Ceredigion, UK, which is located within 2.5 km of each study habitat. At Farm B, rainfall data were measured by a HOBO RG3 Data-Logging Rain Gauge (Tempcon Instrumentation, West Sussex, UK) whilst temperature data were measured by a LogTag Trix-16 Data Logger (LS Technology, Dorset, UK). These instruments were located within 1.5 km of each study habitat. Water pH was measured for each sample using an 8100 pH and Temperature Meter Kit (Omni Instruments LTD, Dundee, UK).

Two 500 mL water samples were collected from each habitat during each sampling visit where sufficient water was available and immediately taken to the laboratory for processing. For each sample, ten 50 mL sub samples were collected from the surface of each small water habitat at random locations with surface water sampled to limit the presence of soil particles which can inhibit molecular analysis. Each 500 mL sample was allowed to settle in the laboratory for 30 min to sediment debris that could clog filters and inhibit molecular analysis, before the water samples were filtered through 2.7 *μ*m micro-glass fibre filters (Whatman, Maidstone, UK) using an electrical suction pump, Büchner flask and a funnel, as described by Jones *et al*. (2018). Previous research has demonstrated that for turbid water samples, as was seen in this study, a larger filter pore size is optimal for maximising total eDNA captured (Thomas *et al*., 2018). A blank control, which consisted of 500 mL of distilled water that was transported, processed and stored alongside collected samples, was analysed for each day of eDNA collection. Filters were stored at −20°C until analysis. Non-disposable equipment was soaked in 7% sodium hypochlorite overnight, before being rinsed in water and dried to avoid cross-contamination between sample collections. DNA was extracted from filter samples using the DNeasy^®^ PowerSoil^®^ kit (Qiagen, Hilden, Germany). All filters, including blank controls, were homogenized using a sterile pipette tip with each whole filter subjected to DNA extraction *via* the PowerSoil^®^ kit protocol. PCR targeting a 288 bp strand of the *G. truncatula* ITS2 gene and gel electrophoresis were used to identify *G. truncatula* eDNA in extracts as previously described by Jones *et al*. (2018) with the modifications stated by Davis *et al*. (2020). The limit of detection of this PCR assay is 0.5 pg *μ*L^−1^ (Davis *et al*., 2020) and the assay has been shown to be capable of detecting *G. truncatula* eDNA in 100% of habitats where the snail was surveyed (Jones *et al*., 2018).

### Data analysis

The *κ* coefficient analysis was undertaken to assess the agreement between positivity of replicates in each sample using SPSS v.27 (IBM, 2020). Factors associated with the detection of *G. truncatula* eDNA were identified using a generalized estimation equation (GEE) model, created in SPSS v.27, where the dependent variable was the presence or absence of *G. truncatula* eDNA in each sample collected. GEE models account for potential correlations within subject, which occurred in this data set as multiple samples collected across sequential time points were nested within the subject (habitat). GEE can also account for missing data points, which also occurred within this data set as not all habitats had water to sample at all sampling points. The working correlation matrix for data inputted into each GEE model was set as AR1, as the lowest Quasi Likelihood under Independence Model Criterion (QIC) values were associated with models created when this working correlation matrix was specified (Cui, 2007). Candidate models were built using a stepwise backward elimination procedure, where the variables with the highest non-significant *P* values (*P* > 0.05) were sequentially removed. Main effect variables offered during model creation included habitat type (pasture or watercourse), snail presence, water pH, mean temperature (°C) and rainfall (mm) on sampling day, mean daily temperature and rainfall 3 days prior to sampling, sampling month and proportion of weeks in study period where sampling water was present, as were biologically plausible interaction effect variables. Farm identity was also included as a fixed factor in each model to account for potential differences between *G. truncatula* snail populations on each farm. The final models were selected *via* their QIC values (Pan, 2001; Burnham and Anderson, 2002), with the models with the smallest QIC regarded as having the best fit.

## Results

**Fig. 2.**
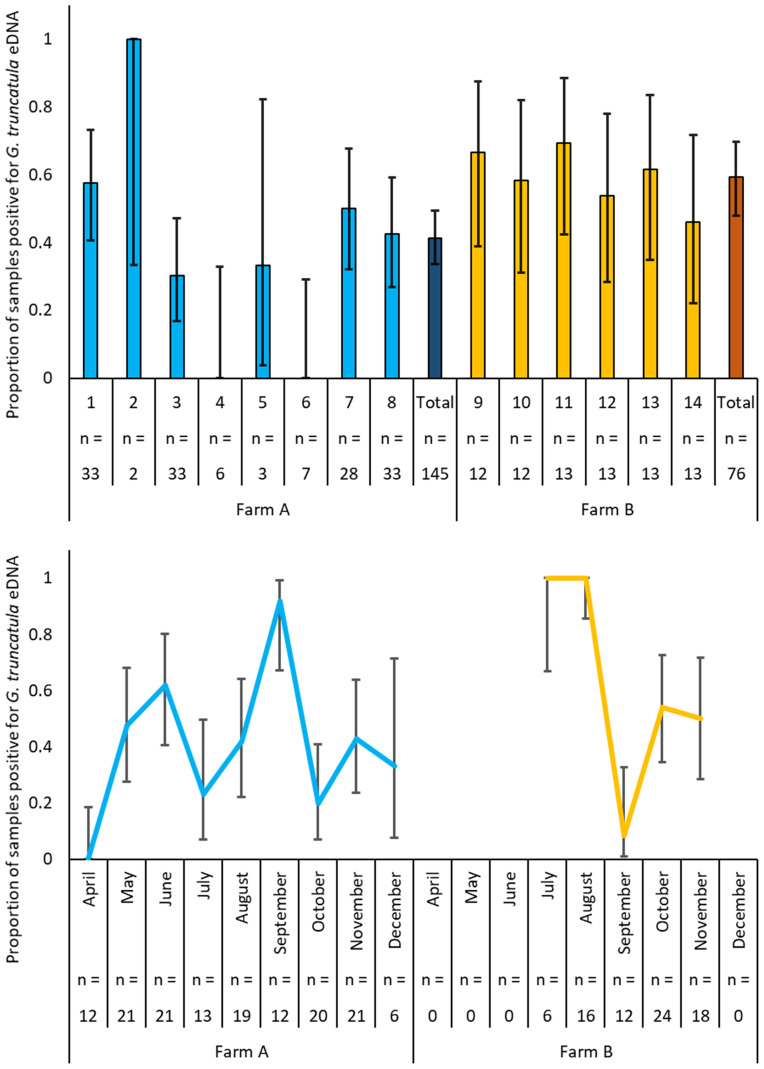
Mean proportion of samples positive for *G. truncatula* eDNA in study habitats (top) and in study months (bottom) across Farm A and B. Error bars denote 95% confidence intervals which were calculated using the proportion confidence intervals function in SPSS v.27.

In total, 221 samples were collected across 14 habitats during the study, 48% of which were positive for *G. truncatula* eDNA. A breakdown of sample positivity rates across each habitat and month can be seen in Fig. 2. Overall, a higher proportion of samples were positive for *G. truncatula* eDNA on Farm B (Fig. 2). Of the 442 replicate samples collected, 35% were positive for *G. truncatula* eDNA, with a significant moderate agreement between replicates for each sample (*κ* = 0.479, *P* < 0.001).

Results of binary GEE analysis of factors associated with *G. truncatula* eDNA presence can be seen in Table 2. Detection of *G. truncatula* eDNA was increased in samples collected when temperatures were higher (*P* < 0.001) and when sample water pH was lower (*P* = 0.047). Samples from habitats where the presence of *G. truncatula* had been confirmed visually during the study period were significantly associated with *G. truncatula* eDNA presence (*P* = 0.006). A significant interaction effect on the number of positive replicates between farm, habitat type and mean 3-day rainfall prior to sampling was also observed (*χ*^2^ = 56.582, d.f. = 4, *P* < 0.001). In this interaction effect, mean 3-day rainfall pre-sampling was significantly positively associated with *G. truncatula* eDNA presence in watercourse habitats on Farm A (*P* = 0.001). Mean 3-day rainfall prior to sampling was significantly negatively associated with *G. truncatula* eDNA presence on Farm B (*P* < 0.001). There was no significant association (*P* = 0.651) between mean 3-day rainfall prior to sampling and *G. truncatula* eDNA presence in pasture habitats on Farm A.

**Table 2.** Best fit binary GEE of environmental and climatic factors associated with *G. truncatula* eDNA detection in 14 habitats during the study period

Parameter	*B*	s.e.	*χ*^2^	Sig.
Farm B	3.308	0.472	49.091	<0.001
Av. 3-day rainfall (mm) prior to sampling				
Watercourse				
Farm A	0.208	0.061	11.485	0.001
Farm B	−0.303	0.051	35.652	<0.001
Pasture				
Farm A	0.020	0.045	0.205	0.651
Farm B	−0.215	0.0431	24.887	<0.001
Av. Temperature °C (sampling day)	0.114	0.026	19.380	<0.001
Confirmed *G. truncatula* presence	0.621	0.226	7.529	0.006
Water pH	−0.922	0.464	3.952	0.047

*B*, *β* coefficient.

## Discussion

The successful application of eDNA analyses on farms requires an understanding of the dynamics of eDNA concentration across space and time and how these interact with local environmental factors (Harrison *et al*., 2019). This study is the first to analyse climatic and environmental factors associated with the longitudinal detection of eDNA of a trematode intermediate snail host in small water body habitats, and reports on factors that should be accounted for when interpreting eDNA analysis results. Of the 14 habitats in this study, *G. truncatula* eDNA was detected in 12, although eDNA detection was not consistent across all timepoints. eDNA is present in very small quantities in the environment, especially the eDNA of species that are likely to expel low volumes of DNA and that are present in low densities (Thomsen and Willerslev, 2015). Steps must therefore be taken to maximize the probability of detecting eDNA, which should include optimizing sampling protocols and timing based on environmental conditions (Rees *et al*., 2014).

The probability of detecting eDNA may also be impacted by a habitat's exposure to a range of environmental and climatic conditions. Unlike many other studies, where pH was positively associated with eDNA presence and concentration (Strickler *et al*., 2015; Seymour *et al*., 2018), this study identified a negative association between pH and *G. truncatula* eDNA detection. Acidic conditions are known to accelerate DNA degradation (Harrison *et al*., 2019). However, in this study, mean pH was relatively neutral (pH = 6.35 ± 0.54 s.d.) and highly acidic conditions, known to have a significant effect on DNA degradation (Strickler *et al*., 2015), were not observed at any study habitat. The negative association observed may therefore be a consequence of the relationship between *G. truncatula* snail presence and environment pH, although contradictory findings towards the snail's preference for either a slightly acidic or neutral pH are present in the literature (Urquhart *et al*., 1996; Charlier *et al*., 2014; Dreyfuss *et al*., 2018). However, as soil pH tends to be lower in areas exposed to heavy rainfall (Brady and Weil, 2002), it is feasible that habitats exhibiting a slightly acidic pH may be better suited to *G. truncatula* due to their humid conditions. A positive relationship was observed between temperature and eDNA detection in this study, which contradicts the known effect of warmer temperatures in accelerating eDNA degradation (Strickler *et al*., 2015; Jo *et al*., 2019). However, higher temperatures are known to increase reproduction rates, population size, activity and mucus, cell and faecal shedding all of which are likely to increase eDNA concentrations in habitats (Stewart, 2019). For *G. truncatula*, it has been shown that general activity, feeding, growth, reproduction rate and lifespan were all significantly greater at 16–22°C compared to 5°C (Hodasi, 1976).

The association between pre-sampling rainfall and eDNA detection was variable between habitat type and location in this study. On Farm A, rainfall was positively associated with eDNA detection in watercourse habitats, whilst it was negatively associated with eDNA detection on Farm B in watercourse and pasture habitats. One of the main differences between watercourse habitats on Farm A and Farm B was their gradient (Table 1), which was greater on Farm B due to local topography. Watercourse gradient will influence water flow rate, particularly following heavy rainfall (Fremier *et al*., 2019). On Farm B, rainfall may therefore have led to eDNA being washed away at a faster rate, leading to reduced eDNA at the sampling sites. In a study by Staley *et al*. (2018), a heavy rainfall event led to a reduction in overall eDNA abundance in water samples collected from creeks due to this wash away effect. Similarly, Jane *et al*. (2015) demonstrated that watercourse flow rate negatively influenced eDNA concentrations at source. However, an increase in overall eDNA species diversity may be seen following heavy rainfall (Staley *et al*., 2018). This would indicate that although large quantities of eDNA may be washed away, some eDNA may also be transported from other areas following heavy rainfall. eDNA detection in the gradually sloped watercourse habitats on Farm A may have benefited from this effect, with rainfall leading to the homogenization of eDNA, initially confined to small usually stagnant water pools on the edges of the watercourse, across the habitat (Pont *et al*., 2018). Furthermore, as feces and associated eDNA are known to sink to the bottom of water columns (Turner *et al*., 2015), which may limit its capture when surface water is sampled (Kamoroff and Goldberg, 2018), rainfall may potentially cause turbulence, lifting eDNA from sediments and limiting sinking (Wotton and Malmqvist, 2001). Ultimately, rainfall is necessary for eDNA surveying of small water body habitats, and without sufficient water levels, sampling may not be possible as was the case in multiple timepoints across most habitats in this study which dried up during the summer months. *Galba truncatula* snails are able to survive these dry conditions across the summer months before remerging to potentially shed infective cercariae (Dreyfuss *et al*., 2015), and thus eDNA surveys must be timed to ensure samples can be collected.

Considering the complex nature of the relationship between eDNA detection and rainfall seen in this study, the reliability of eDNA detection as an accurate indication of species presence at a particular sampling site may be questioned, especially in watercourse habitats where eDNA can be transported vast distances with the flow of water (Pont *et al*., 2018). However, a study by Wilcox *et al*. (2016) demonstrated that the median transport distance of detectable fish eDNA in small streams was between 74 and 145 m, with the distance transported increasing as water flow rate increased. Studies have demonstrated that eDNA transport in large rivers may be over 100 km (Deiner *et al*., 2016; Pont *et al*., 2018), however considering the nature of *G. truncatula* watercourse habitats, which are commonly small slow-moving streams and drainage ditches, eDNA transportation down-stream should be limited. Watercourses are also potential movement corridors for *G. truncatula* snails, with snails capable of either migrating up stream over 100 m a month in some instances (Rondelaud, 1983) or being washed down stream following heavy rainfall (Rondelaud *et al*., 2005). In this instance, detecting eDNA a few hundred meters from the original source may not be a major issue as it would fall within the range of potential *G. truncatula* migration.

Two replicate water samples of 500 mL were collected at each sampling point in this study and increasing number of replicates and the sample volume would likely have increased detection rates in this study. According to Schmidt *et al*. (2013), the probability of detecting target species' eDNA in a sample increases logarithmically with the number of replicate samples collected, with the probability of detecting the eDNA of their target species above 95% only when six replicate samples were collected. Furthermore, Mulero *et al*. (2020) found that 3 L of water was required to be sampled for the accurate detection of *Schistosoma* intermediate snail host *B. truncatus.* However, increasing replicate numbers and sampled water volume can be challenging. Each replicate adds cost to the protocol (Sengupta *et al*., 2019), which may become prohibitive if multiple habitats need to be assessed on a site. Furthermore, water from small water habitats often contains various debris that can clog filters and limit the water volume that can be sampled (Thomas *et al*., 2018).

*Galba truncatula* eDNA was significantly more likely to be detected in habitats where *G. truncatula* snails were surveyed during the study period; however, the density of snails surveyed at each visit was not a significant factor associated with eDNA detection, although this could be explained by the comparatively unsensitive and variable nature of traditional *G. truncatula* surveys which may fail to identify snails present in mud, water or thick vegetation. This may also explain why *G. truncatula* eDNA was detected in habitats where these snails were not discovered *via* traditional surveying methods during the study period, a similar finding to that of Jones *et al*. (2018). This would highlight the sensitive nature of eDNA assays, which can discover the presence of species when present at very low densities such as *G. truncatula*. Detecting the presence of intermediate snail hosts, even in low-density populations may be vital considering that a small number of snails are capable of shedding thousands of infective trematode stages into the environment (Dreyfuss *et al*., 2015). However, it must be noted that false positives may be caused by multiple factors including eDNA persistence following the disappearance of a species, transport of eDNA from another habitat, DNA contamination during the sampling/analysis process and amplification of another species' DNA (Ficetola *et al*., 2016). Steps may be taken to limit DNA contamination (Goldberg *et al*., 2016) and to ensure assay specificity (Davis *et al*., 2020); however, the potential for eDNA to be transported to the sampling site or its persistence following previous inhabitation cannot be controlled. The calculation and incorporation of false-positive probability rates into site occupancy models has been suggested as a method for improving eDNA survey results (Ficetola *et al*., 2016) and may be vital when surveying across a wide range of habitat types that certain intermediate snail hosts can inhabit. For example, the target of this study *G. truncatula* can inhabit permanent wetlands, spring heads, ponds, streams, ditches and poached soils on pastures in both temperate and arid climates (Dreyfuss *et al*., 2015). Indeed, successfully contextualising eDNA presence or absence in habitats of varying type which are exposed to a wide range of environmental and climatic conditions will be vital for practical application of eDNA surveying in the field of parasite control. Extensive further research into eDNA dynamics is therefore of upmost importance to enable the use of these tools in the future.

In summary, this study identifies environmental and climatic factors associated with the detection of the trematode intermediate snail host, *G. truncatula*, in small water body habitats over time. These factors should be considered when devising standardized eDNA sampling protocols and data analysis to ensure eDNA surveying goals are robustly met. Future research should build on knowledge gained in this study to use and test site occupancy models for identifying *G. truncatula* and other intermediate snail hosts in small water body habitats.

## Data Availability

Data are available from the corresponding author upon reasonable request.
